# Interfacial Properties of Surface-Modified Lignin Nanoparticles

**DOI:** 10.1021/acs.langmuir.5c01244

**Published:** 2025-07-04

**Authors:** Mina Zare, Patrícia Figueiredo, Maarit H. Lahtinen, Kristiina S. Hilden, Sami Hietala, Kirsi S. Mikkonen

**Affiliations:** † Department of Food and Nutrition, Faculty of Agriculture and Forestry, 3835 University of Helsinki, 00014 Helsinki, Finland; ‡ Helsinki Institute of Sustainability Science (HELSUS), University of Helsinki, 00014 Helsinki, Finland; § Department of Microbiology, Faculty of Agriculture and Forestry, University of Helsinki, 00014 Helsinki, Finland; ∥ Department of Chemistry, University of Helsinki, 00014 Helsinki, Finland

## Abstract

The interfacial properties of surface-modified lignin nanoparticles (LNPs) through the adsorption of various compounds or grafting of different ligands dictate their uses and potential applications in life sciences. In this study, we investigate softwood (Lignoboost lignin, LB) and hardwood (Birch lignin, BB) LNPs and compare them to LNPs treated with laccase-induced oxidation, hemicellulose (galactoglucomannan (GGM) and glucuronoxylan (GX)) adsorption, and laccase-induced hemicellulose adsorption. Then, we compare the interfacial properties of the obtained particles through contact angle and interfacial tension analyses and compare these to the stability of their emulsions. The study reveals that hemicellulose adsorption increases the hydrophilicity of LNPs, except for GX, and laccase-induced hemicellulose adsorption increases the hydrophobicity of particles. The unique features of functionalized BB-LNPs with GX are that they adsorb rapidly at the interface, show increased hydrophobicity, and avoid aggregation in the emulsion. The roles of hemicellulose adsorption and laccase-induced hemicellulose adsorption are discussed by considering the interfacial tension and contact angle for emulsification. These findings lay the groundwork for comprehending the role of hemicellulose in surface-modified LNPs in interfacial stabilization and expand their potential uses in food, pharmaceuticals, and biomedical sciences.

## Graphical abstract

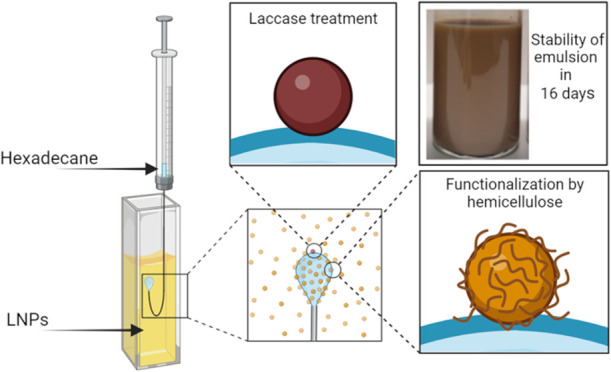

## Introduction

Lignin, the second most abundant renewable resource and the most prevalent biopolymer based on aromatic units, is isolated from lignocellulosic biomass and exhibits high valorization potential.^1^ Recently, lignins have garnered increased attention from the research community due to their unique features, including antioxidant and antimicrobial properties, UV-blocking ability, biodegradability, and biocompatibility, showcasing their potential for advanced applications.^2^ Several factors influence the properties of lignin, such as the plant species and the isolation method used. Additionally, lignin is composed of methoxylated polyphenols, and its basic monomeric units, guaiacyl (G), syringyl (S), and p-hydroxyphenyl (H), can vary based on the source.^3,4^ The valorization of lignins has been hampered by their complex and heterogeneous molecular structure.

One approach to overcome these challenges involves transforming lignin extracted from plant biomass into lignin nanoparticles (LNPs) and functionalizing them. Surface modification of LNPs holds the potential to expand their applications in life sciences. This can be achieved by the adsorption or grafting of different ligands, such as polysaccharides, proteins, peptides, synthetic polymers, small molecules, *etc.* To maintain their green character, it is essential to employ sustainable methods and materials during the functionalization of LNPs. Grafting methods reported so far commonly use harsh solvents and reagents and/or employ high temperatures.^5^ For the first time, we have proposed the facile functionalization of lignin with green surface modification at room temperature in a single step through spontaneous hemicellulose adsorption.^6^

Hemicelluloses are flexible carbohydrate molecules whose conformational structures in aqueous or physiological environments can be influenced by interactions with neighboring ions or molecules, particularly through explicit hydration.^7^ They have complex structures that vary among different wood species. In softwoods, the primary polysaccharide is galactoglucomannan (GGM), whereas in hardwoods, glucuronoxylans (GX) are the predominant hemicelluloses.^8–10^ Hemicelluloses are side streams of the wood industry and may also contain lignin moieties with phenolic hydroxyl groups.^11,12^ Laccases are enzymes that oxidize phenolic and aromatic compounds with the concomitant reduction of molecular oxygen to water. They are studied for polymerizing or depolymerizing lignin and phenols, and the reactions can be influenced by factors such as redox mediators, substrate, laccase type, pH, and dosage.^3,4^ Therefore, laccases can provide opportunities for lignin valorization through cross-linking or depolymerization, with the properties of LNPs as substrates for laccases being influenced by the variation of monomeric units and functional groups. In a previous study by Figueiredo et al., the enzymatic treatment of LNPs using fungal laccases was reported,^3^ while in this study, we extend the work by laccase-induced hemicellulose adsorption to LNP surface. Functionalizing LNPs renders them suitable for various life science applications, such as pharmaceuticals, food science, and hydrocolloids. Possible uses include serving as emulsion stabilizers, dispersants, drug delivery carriers, bioactive compound encapsulants, or even rheology modifiers to enhance the texture and stability of products. By modification of the LNP surface, their potential as versatile functional materials for different industrial and biomedical applications can be expanded.

Hydrocolloids and nanoparticles have become crucial constituents in many life science applications, such as emulsions.^13^ The adsorption of LNPs at the liquid–liquid interface plays a critical role in emulsion stabilization and targeted delivery in biomedicine.^14^ Emulsions have been widely used to protect, transport, and target the delivery of bioactive compounds.^15^ The wettability and adsorption of nanoparticles at the oil–water interface are the key points to having a stable emulsion for different applications. The utilization of GGM and GX as emulsion stabilizers has been well investigated, and the studies reported that both GGM and GX have a good capacity to stabilize oil-in-water emulsions, and residual lignins brought additional emulsion stability functionality.^11,16,17^ However, the interfacial behavior of functionalized LNPs using hemicellulose as an adsorbed surface component has not been studied before. Exploring this could provide insights into the Pickering emulsion model, where solid particles such as LNPs act as stabilizers by adsorbing at the oil–water interface, offering the potential for enhanced stability and functionality in various industrial and biomedical applications.

Exploring the functional properties and interfacial phenomena of surface-modified LNPs is a significant scientific endeavor with broad implications across various industries. This fundamental research aims to examine the functional properties of surface-modified LNPs through two methods of hemicellulose adsorption: spontaneous and laccase-induced. It seeks to understand interfacial phenomena by studying the solid–gas interface, examining the liquid–liquid interface, and analyzing their behavior in an oil/water system.

We elucidate the impact of surface modification of hardwood and softwood LNPs based on their interfacial and wettability properties, along with the Pickering emulsion model. These methods, with their distinct adsorption mechanisms, warrant further study to optimize industrial applications and enhance the performance and stability of biobased materials. This research not only promotes the utilization of renewable resources but also advances material science and supports sustainable development practices. By understanding and harnessing the unique properties of modified LNPs, this study has the potential to lead to innovative applications and contribute to a more sustainable future.

## Results

### Size and ζ-Potential of LNPs

The size and ζ potential of LNPs contribute strongly to the particle behavior at interfaces. They influence the mobility, affinity, and arrangement of LNPs at the oil–water interface, which further affects the interfacial tension. The ζ potential is the electrical potential at the slipping plane within the interfacial double layer of a colloidal particle. It reflects the effective surface charge and serves as an indirect indicator of the strength of electrostatic repulsion between particles in a dispersion.^18^ Our results revealed that the source of lignins and the isolation method affect the size and zeta potential of LNPs (Figure S1a,b). Dynamic light scattering showed that LB-LNPs had a smaller particle size (∼100 nm) compared to BB-LNPs (>150 nm). The prepared LNPs demonstrated polydispersity index (PDI) values below 0.16, indicating their homogeneity and monodispersity.

Table 1 summarizes the ζ-potential and average particle sizes of LB-LNPs and BB-LNPs before and after functionalization with GX and GGM. Both types of LNPs were negatively charged. The ζ-potential of functionalized LB-LNPs with GX and GGM increased from −41.6 ± 0.5 to −22 ± 1 and −35 ± 0.9, respectively. The GX and GGM increased the ζ-potential of BB-LNPs (−46.3 ± 3.2) to −31.5 ± 0.8 and −34.7 ± 1.9, respectively. The spontaneous adsorption of GX and GGM reduced the average size of LB-LNPs from 111.6 ± 2.8 to 101.3 ± 1.5 and 99.3 ± 0.5, respectively, while they did not change the size of BB-LNPs.

**Table 1:** ζ-potential and Average Particle Size of LB-LNPs and BB-LNPs before and after Functionalization with GX and GGM^a^

**sample**	**ζ-potential (mV)**	**size (nm)**
LB-LNPs	–41.6 ± 0.5	111.6 ± 2.8
LB-LNPs + GX	–22.0 ± 1.0	101.3 ± 1.5
LB-LNPs + GGM	–35.0 ± 0.9	99.3 ± 0.5
BB-LNPs	–46.3 ± 3.2	156.6 ± 2.8
BB-LNPs + GX	–31.5 ± 0.8	158.3 ± 1.5
BB-LNPs + GGM	–34.7 ± 1.9	155.3 ± 2.3

a LB-LNPs (Lignoboost lignin nanoparticles), LB-LNPs + GX (Lignoboost lignin nanoparticles functionalized with GX), LB-LNPs + GGM (Lignoboost lignin nanoparticles functionalized with GGM), BB-LNPs (Birch lignin nanoparticles), BB-LNPs + GX (Birch lignin nanoparticles functionalized with GX), and BB-LNPs + GGM (Birch lignin nanoparticles functionalized with GGM).

### Contact Angle Studies

The contact angle (Θ) is defined as the angle formed between the tangent to a liquid droplet at the three-phase contact point (liquid–solid–air interface) and the surface of the substrate. The contact angle is commonly used to evaluate the wetting behavior of a liquid on a surface: a low contact angle indicates strong wetting (hydrophilic behavior), while a high contact angle signifies poor wetting (hydrophobic behavior).^19^ To analyze the contact angle of coated LNPs on a silicon wafer surface, we needed to ensure uniform coverage of the LNPs on the wafer. A double layer of 1 mg/mL LNPs provided complete coverage of the silicon wafer surface and was used in subsequent analyses. AFM images, captured at various random locations for each sample, confirmed the uniformity of the thin films (Figure S2). However, due to scanning artifacts—likely caused by tip damage—the lignin nanoparticles did not appear spherical, as previously confirmed in our recent study.^6^ The contact angle measurement results showed that LB-LNPs were more hydrophilic than BB-LNPs (Figure 1). The contact angle of LB-LNPs was 34.2 ± 1.5 mm, and that of BB-LNPs was 39.5 ± 1.9. Our experimental results reveal differences in the hydrophobicity/hydrophilicity of LNPs functionalized with GX and GGM. After hemicellulose treatment, the contact angle of LB-LNPs decreased, indicating increased hydrophilicity, with measurements of 30.2 ± 0.1 for GGM and 30.8 ± 1.4 for GX, respectively. In contrast, the contact angles of BB-LNPs were not consistent; GGM treatment reduced the contact angle to 34.2 ± 0.9, which indicates increasing hydrophilicity, while GX treatment increased the contact angle to 47.5 ± 2.3, which indicates increased hydrophobicity.

**Figure 1:**
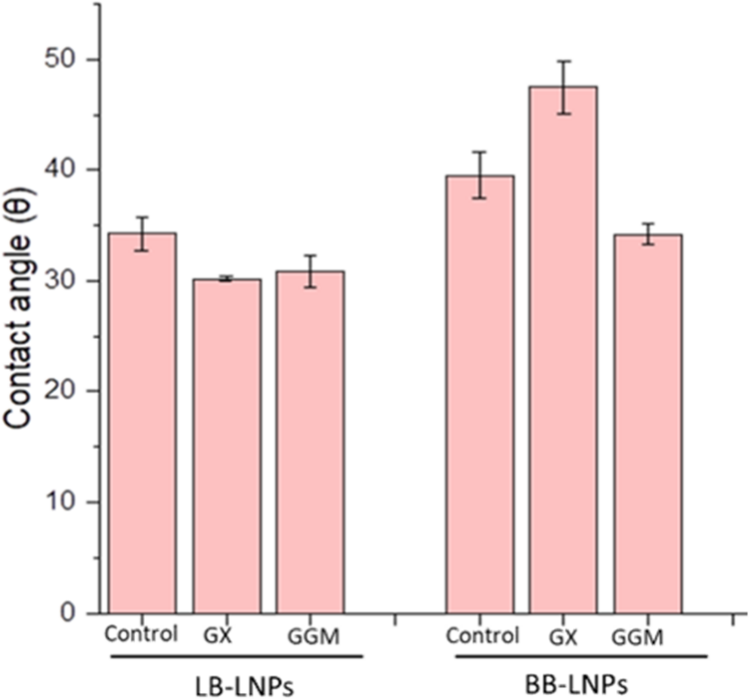
Contact angle of lignin nanoparticles (LNPs) and LNPs functionalized with GX and GGM. Refer to Table 1 for abbreviations.

### Contact Angle of Functionalized LNPs via Laccase-Assisted Hemicellulose Adsorption

The hydrophobicity/hydrophilicity of laccase-treated LNPs (using *Ds*Lcc4) and hybrid LNPs is depicted in Figure 2. A noticeable increase in hydrophobicity for the laccase-treated LB-LNPs compared with BB-LNPs is found. Specifically, the contact angle of LB-LNPs increased from 34.2 ± 1.5 to 50.8 ± 1.6° after laccase treatment. For LB-LNPs functionalized with GX using laccase, the contact angle rose from 30.2 ± 0.1 to 41.2 ± 2.1°, and for those functionalized with GGM, it increased from 30.8 ± 1.4 to 44.1 ± 2.8°. The laccase treatment increased the contact angle of BB-LNPs from 39.5 ± 2.1 to 40.7 ± 2.2°. For BB-LNPs functionalized with GX and GGM using laccase, the contact angles increased to 49.6 ± 1.1 and 41.3 ± 2.3°, respectively, from initial values of 47.5 ± 2.3 and 34.2 ± 0.9°. Laccase-assisted functionalization with GGM increased hydrophobicity more than that of the control (BB-LNPs) and BB-LNPs functionalized with GX.

**Figure 2:**
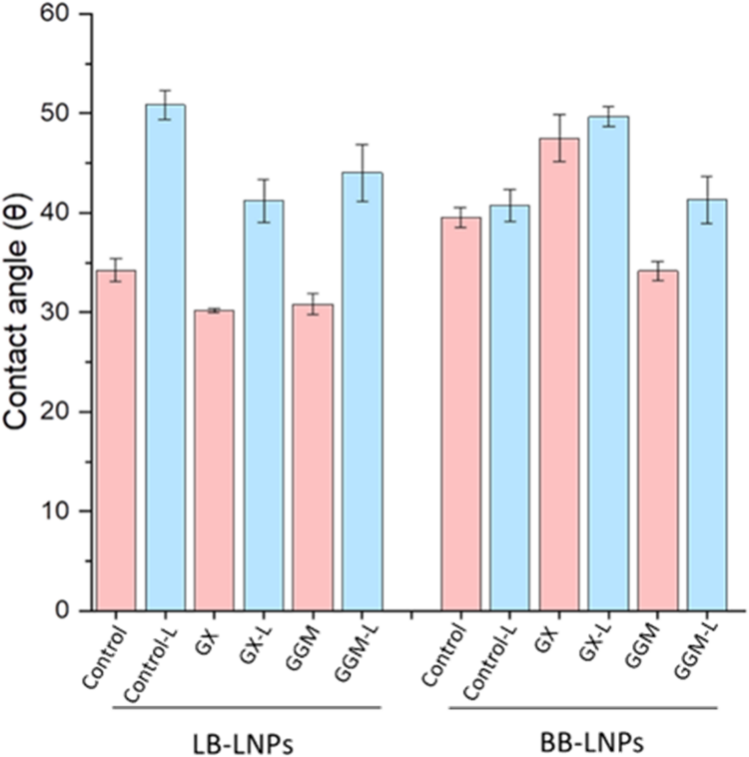
Contact angle of lignin nanoparticles (LNPs) and hybrid hemicellulose-LNPs with *Ds*Lcc4 laccase and without laccase treatment. Refer to Table 1 for abbreviations.

### Interfacial Tension (IFT)

The interactions of LNPs in oil/water systems are fundamental for applications such as Pickering emulsions. Interfacial tension (IFT) indicates the spontaneous adsorption of components between a continuous liquid phase and droplet surface. IFT of LNPs and LNPs treated with hemicelluloses (GGM and GX) in an oil/water system are presented in Figure 3 and detailed in Figure S3. A reduction in IFT with both LB-LNPs and BB-LNPs is observed compared to the control (H_2_O). However, BB-LNPs demonstrated a more substantial decrease in IFT compared to LB-LNPs. IFT measurements showed a decrease from 43.2 ± 0.2 mN/m in a control system to 41.6 ± 0.3 and 31.1 ± 0.2 mN/m for LB-LNPs and BB-LNPs at 60 min and 20 s, respectively, in an *n*-hexadecane/water system. At this point, the reaction appeared to have reached a steady state, indicating that equilibrium had been established.

**Figure 3:**
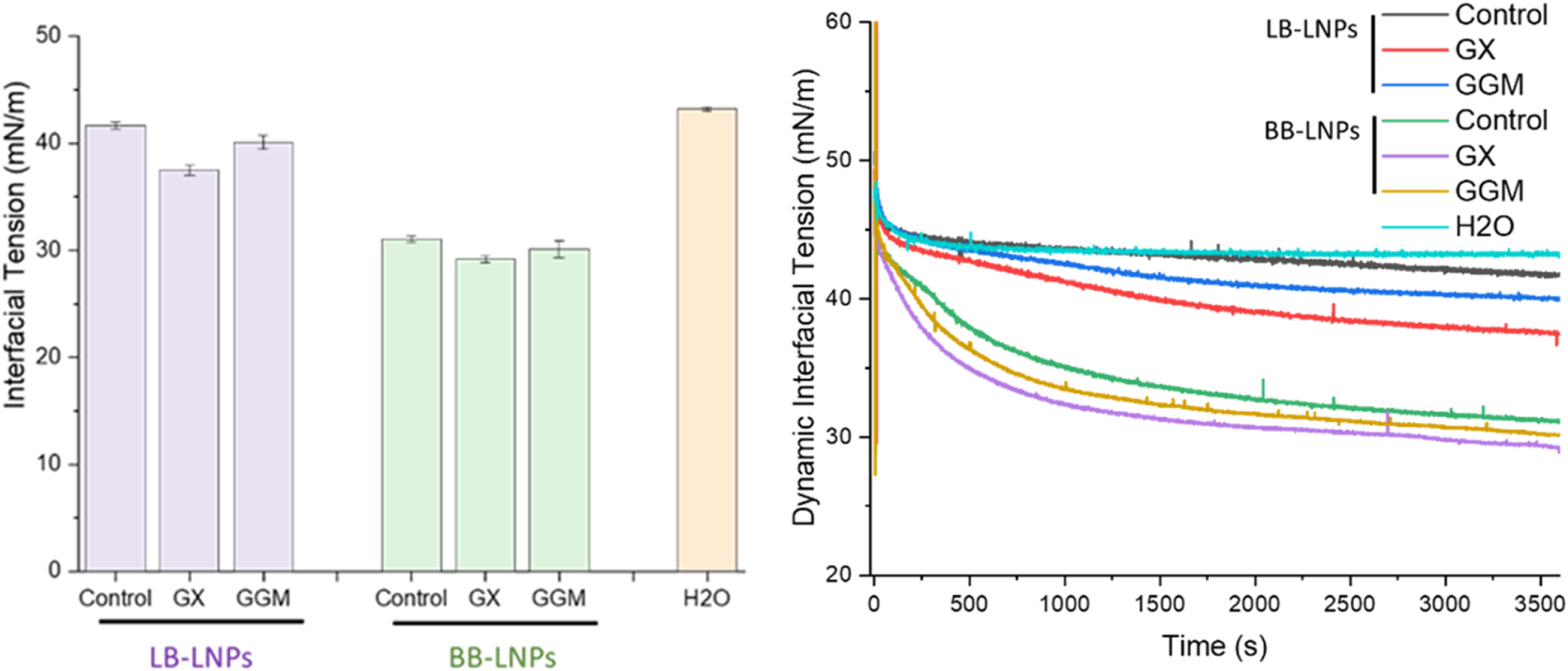
illustrates the interfacial surface tension of 0.05 mg/mL of LB-LNPs, BB-LNPs, and hybrid hemicellulose-LNPs at 60 min and 20 s. H_2_O is an oil in water without any nanoparticles. Refer to Table 1 for abbreviations.

The IFT of LB-LNPs functionalized with GX and GGM was 37.5 ± 0.4 and 40.1 ± 0.6 mN/m, respectively. The IFT of BB-LNPs functionalized with GX and GGM was 29.1 ± 0.3 and 30.1 ± 0.8 mN/m, respectively. In examining the impact of hemicellulose functionalization on LNPs, our studies revealed that functionalization with GX affected the IFT more than GGM. This result is reflected in a faster reduction of IFT when LNPs were treated with GX compared to GGM.

IFT of LB- and BB-LNPs treated with five different *in-house* laccases (*C*cLcc9), (*O*rLcc2Mut), (*T*pLccMut), (*D*sLcc4*), and* (*P*rLcc2) are shown in Figure 4. The LB-LNPs remained relatively similar regardless of the use of laccases (Figure 4). Conversely, all tested laccases were observed to increase the IFT of BB-LNPs, yet without notable differences among the types of laccases applied. The outcomes demonstrate that laccase treatment increased the IFT of BB-LNPs more than that of LB-LNPs.^20,21^ *Ds*Lcc4 was chosen for further experiments and characterization.

**Figure 4:**
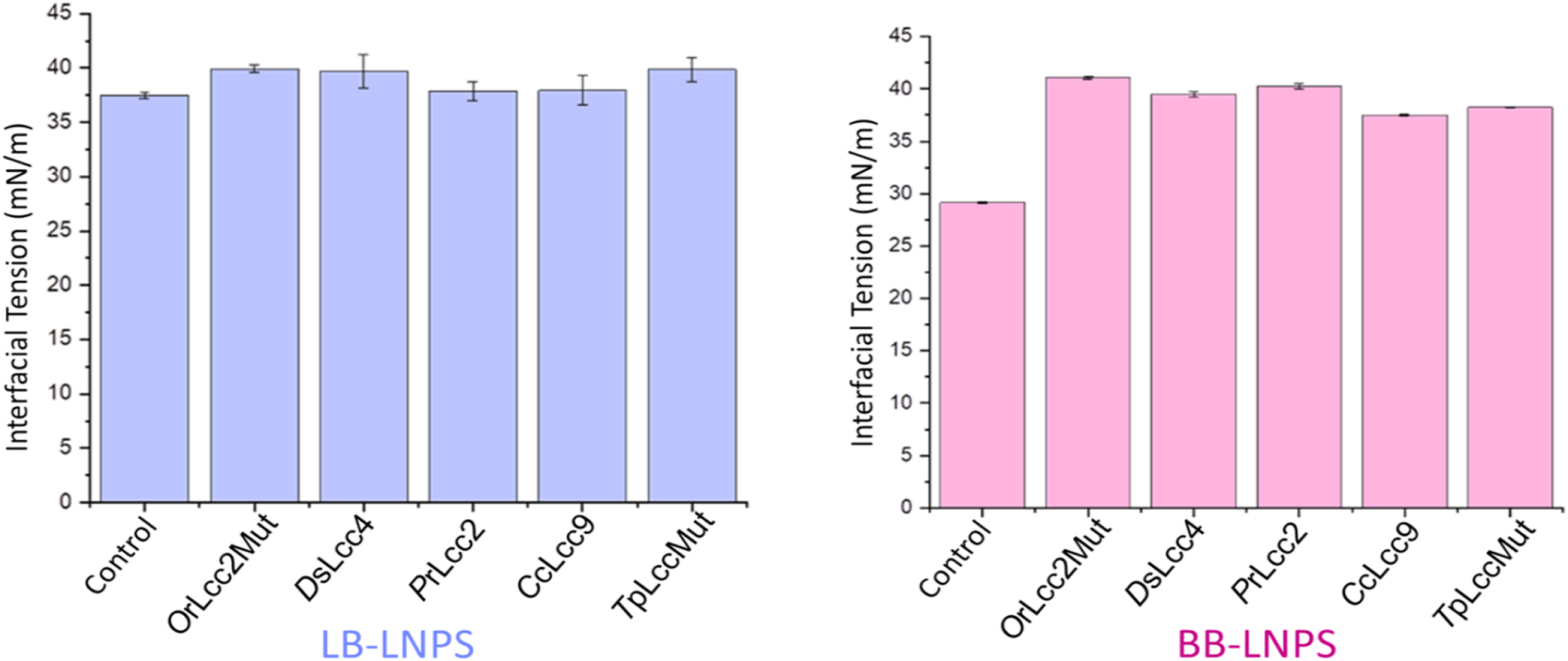
Interfacial tension of laccase-assisted oxidized LB-LNPs and BB-LNPs (0.05 mg/mL), measured at 60 min and 20 s, using five types of *in-house* produced laccases. Lignoboost softwood lignin (LB), hardwood lignin (BB), lignin nanoparticles (LNPs), (*O*rLcc2Mut), (*D*sLcc4), (*P*rLcc2), (*C*cLcc9), and (*T*pLccMut) were used in the study.

The IFT of LNPs after *Ds*Lcc4 laccase-assisted hemicellulose adsorption is depicted in Figure 5. The IFT of LB-LNPs and hybrid LB-LNPs showed only a minor increase with laccase treatment, which was not statistically significant. The IFT of BB-LNPs increased from 31.1 ± 0.3 to 38.4 ± 0.4 after laccase treatment. For BB-LNPs functionalized with GX, the IFT increased from 29.2 ± 0.3 to 37.8 ± 0.2, and for those functionalized with GGM, it increased from 30.1 ± 0.8 to 37.4 ± 0.4, after laccase treatment. The paired *t* test results show a t-statistic of −17.85 and a p-value of 0.0031. Since the p-value is less than 0.05, the difference between the IFT values before and after laccase treatment is statistically significant.

**Figure 5:**
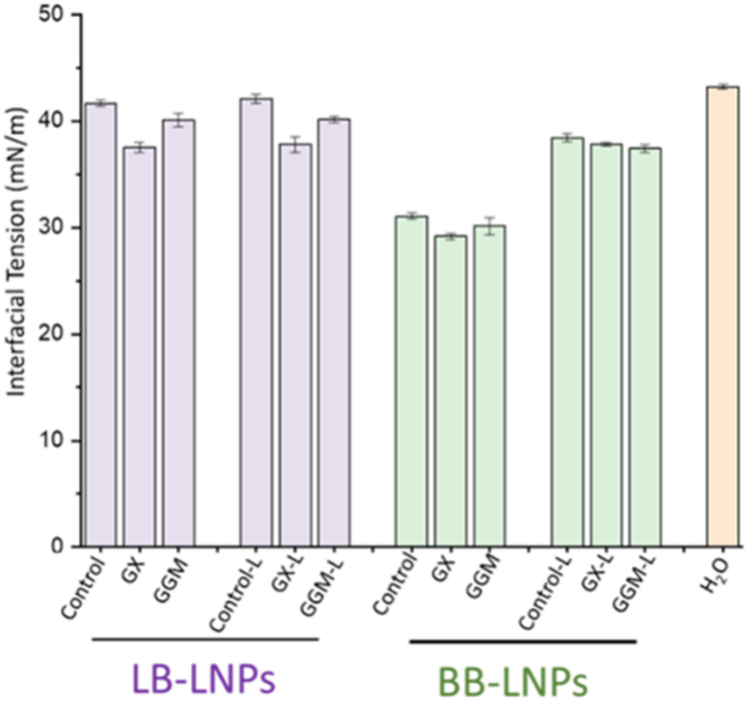
Interfacial tension of hemicellulose-functionalized LNPs with *Ds*Lcc4 laccase and without laccase treatment at 60 min and 20 s. Refer to Table 1 for abbreviations.

### Emulsion Characterization

#### Droplet Size and Its Distribution

The correlation between interfacial tension (IFT) and droplet size in emulsions is critical as it directly influences both emulsion formation and stability. Surface properties—particularly the hydrophobicity or hydrophilicity of lignin nanoparticles (LNPs)—play a significant role in droplet formation by affecting how liquid phases disperse. Therefore, we analyzed droplet size and size distribution to evaluate how these surface properties and the IFT affect emulsion stability. Figure 6 presents the droplet size distribution of different emulsions on days 1 and day 16. Emulsions stabilized by LB-E, LB-GX-E, and LB-GGM-E are shown in Figure 6a. The LB-E emulsion exhibited the largest average droplet size (104.6 μm), while the addition of GX and GGM slightly reduced the size after storage for 16 days of storage. Figure 6b shows the emulsions stabilized by laccase-treated LNPs (LB-L-E) and their GX and GGM hybrids. These emulsions initially showed average droplet sizes around 118 μm with minimal change during the 16-day storage period, indicating moderate stability.

**Figure 6:**
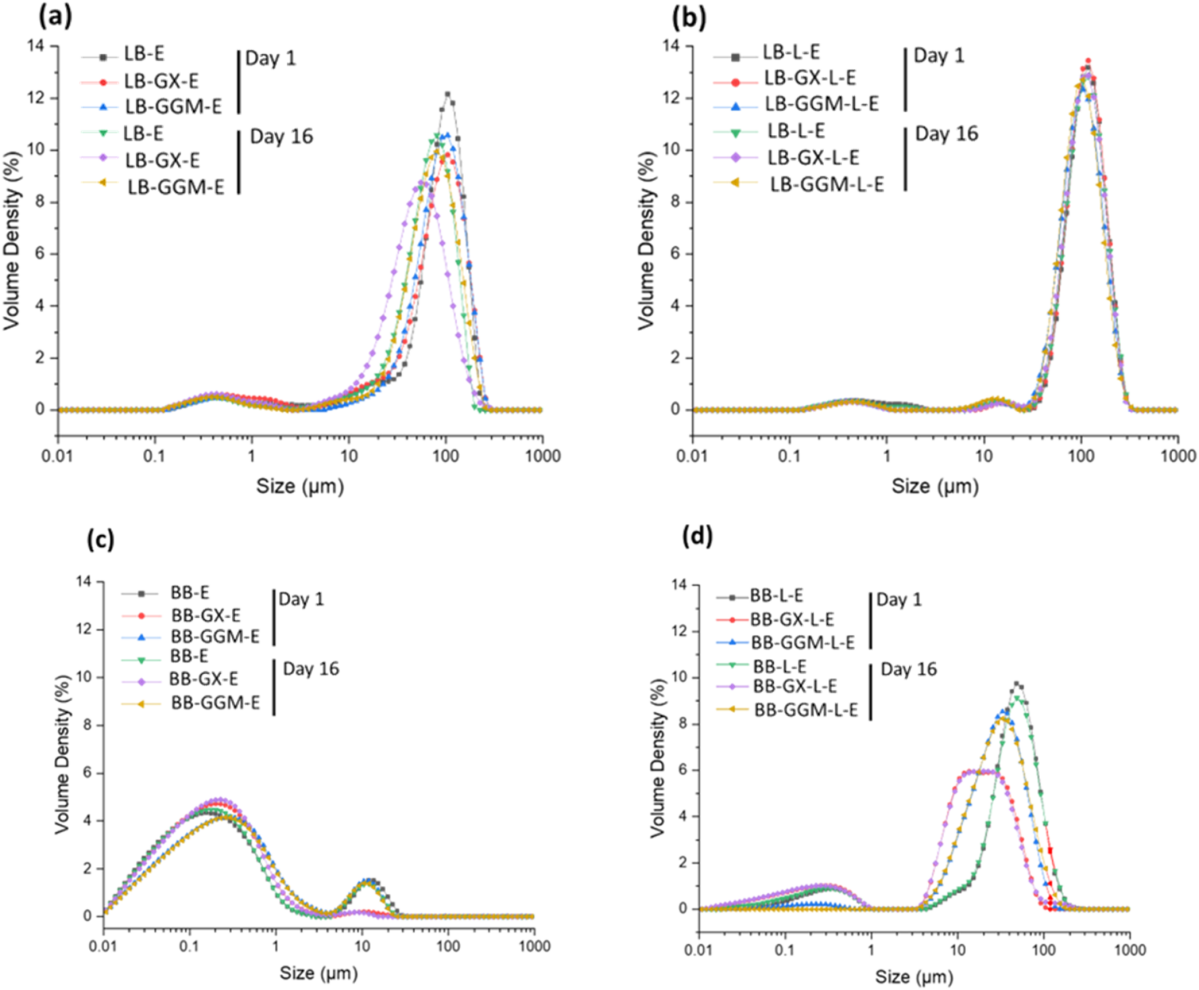
Droplet size distribution of emulsions at day 1 and day 16. (a) LB-E, LB-GX-E, and LB-GGM-E; (b) LB-L-E, LB-GX-L-E, and LB-GGM-L-E; (c) BB-E, BB-GX-E, and BB-GGM-E; and (d) BB-L-E, BB-GX-L-E, and BB-GGM-L-E. Refer to Table 1 for definitions of sample abbreviations.

Droplet flocculation and coalescence were confirmed by optical microscopy (Figure S4), which showed an increased droplet size immediately after preparation in selected emulsions. Figure S5 illustrates the stability of these emulsions over the 16-day period.

Figure 6c shows the size distribution of the emulsions stabilized by BB-E and its GX and GGM hybrids. These emulsions remained stable during storage, with BB-E and BB-GGM-E showing a bimodal droplet distribution (average sizes of 0.2 and 0.3 μm, respectively). BB-GX-E exhibited a monodisperse distribution centered at approximately 0.2 μm. In Figure 6d, the laccase-treated counterparts—BB-L-E, BB-GX-L-E, and BB-GGM-L-E—also showed stable droplet distributions. The average droplet sizes for BB-L-E and BB-GGM-L-E were 48.6 and 33.1 μm, respectively. BB-GX-L-E displayed a broader distribution, with sizes ranging between 13 and 24 μm.

To further analyze the size distribution, both the volume average diameter (D­[4,3]) and the surface-area-weighted diameter (D­[3,2]) were used. The D­[4,3] value reflects the mean droplet size weighed by volume and is particularly sensitive to changes in larger droplets. The D­[3,2] value emphasizes the surface area and is more influenced by the smaller particles. These two parameters helped clarify how surface functionalization affects particle morphology and stability.

The volume average diameters (D­[4,3]) after 16 days are shown in Figure S6a. For LB-E and its GX and GGM hybrids, the D­[4,3] values decreased slightly during storage, suggesting gradual stabilization. BB-E and BB-GX-E remained relatively constant, while BB-GGM-E showed a marked increase from 1.4 to 18.3 μm over 16 days, indicating emulsion instability. Laccase treatment had little effect on the droplet size stability across all samples.

Figure S6b shows the surface-area-weighted diameters (D­[3,2]) of the emulsions. LB-E and LB-GGM-E showed a slight decrease over time, while LB-GX-E remained stable. BB-based emulsions (BB-E, BB-GX-E, BB-GGM-E) all had average droplet sizes around 0.1 μm, which remained unchanged throughout storage. In contrast, LB-E exhibited a much larger D­[3,2] value (>7.0 μm), consistent with its larger droplet size. All laccase-treated BB-based emulsions maintained stable D­[3,2] values except for BB-GGM-L-E, which exhibited a significant increase after 16 days and ultimately failed to stabilize the emulsion. The larger droplet size and instability of BB-GGM-E are consistent with its higher global Turbiscan Stability Index (TSI) value.

#### Stability of Emulsion

The stability of emulsions as well as their stability changes during storage were assessed using the global Turbiscan Stability Index (TSI), where a lower index indicates higher stability (Figure 7). The optical microscopy images of LNP emulsions after preparation are shown in Figure S4. The stability indices for LB-LNP emulsion and hybrid hemicellulose-LB-LNP emulsion were about 70–80 (Figure 7a). The LB-LNPs stabilized emulsion functionalized with GX and GGM exhibited a slight reduction in the TSI values; however, these changes were negligible and did not affect the overall outcome, and the emulsions remained unstable. Laccase treatment of LB-E and hybrid hemicellulose-LB-E reduced the TSI value while they were unstable.

**Figure 7:**
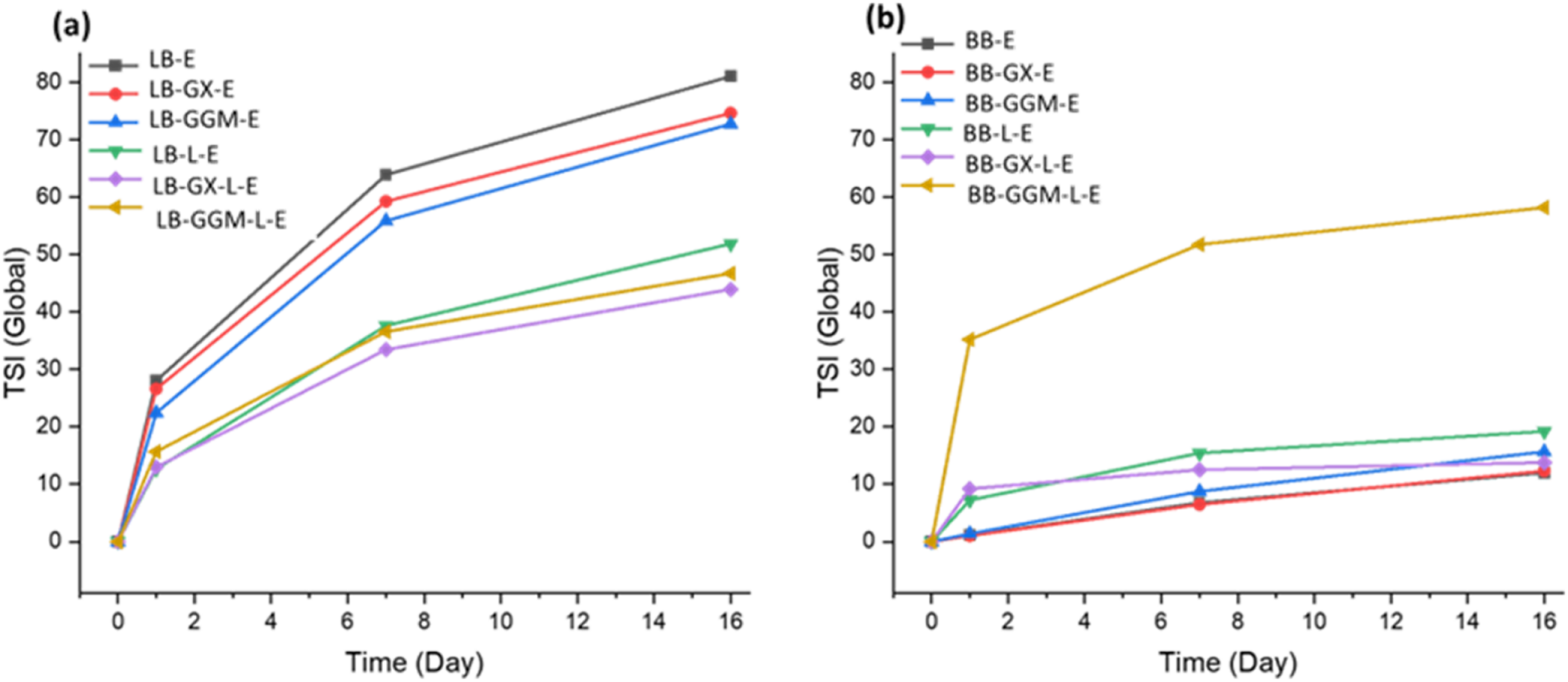
Global Turbiscan Stability Index (TSI) of the emulsions prepared using LB-LNPs, LB-X, LB-GGM, BB-LNPs, BB-GX, and BB-GGM, and measurements on days 0, 1, 7, and 16. Refer to Table 1 for abbreviations.

The TSI values of the BB-E and hybrid hemicellulose-BB-E were approximately 12–16 (Figure 7b), indicating that these emulsions were more stable compared to those stabilized with LB-E and hybrid hemicellulose-LB-E. It was unexpected that the BB-GGM-L-E showed a clear instability, by visualization, and a TSI value of about 58, and all of the droplets were creamed on the top of the emulsion. The emulsion stability of BB-GX-L-E remained almost constant over 16 days of storage, which indicates a stable emulsion.

Optical light microscopy assesses the structural integrity of the droplets, providing insights into the microstructure of emulsions and clues about potential instability and phase separation phenomena through droplet deformation or coalescence, which can critically affect the emulsion’s functionality and appearance. Therefore, optical light microscopy imaging was used to assess the size distribution of the droplets within an emulsion and confirm the occurrence of emulsion stabilizing and breakdown mechanisms, such as creaming, flocculation, and coalescence. The optical light microscopy images of droplets are displayed in Figure S4, and Figure S5 shows the stability of the emulsion over 16 days of storage. The results show aggregation by flocculation and creaming in LB-E and hybrid hemicellulose-LB-E without laccase treatment. The optical microscopy images of LB-L-E and hybrid hemicellulose-LB-L-E display aggregation and coalescence. Interestingly, no aggregation was observed in the BB-E and hybrid hemicellulose-BB-E without laccase treatment. Notably, the lowest coalescence and flocculation among the laccase-treated LNPs was observed in the BB-GX-L-E.

## Discussion

### Plain Lignin Nanoparticles

The hydrodynamic diameter of LB-LNPs was slightly smaller than that of BB-LNPs, likely due to differences in their molar masses. These differences stem from the distinct origins of the two technical lignins and impact noncovalent forces during the self-assembly process.^3,22^ Additionally, the π–π interactions between the guaiacyl units in LB lignin are stronger than those among the syringyl units in BB lignin, resulting in more compact and smaller LB-LNPs. The negative surface charge of LNPs is caused by the highly abundant phenolic, aliphatic hydroxyl, and carboxylic groups on their surface, which can lead to stabilization in colloidal dispersion due to the electric double-layer repulsion.^3,22^

The amphiphilic nature of lignins can stabilize the interface between oil and water phases as they interact with both polar and nonpolar substances.^23^ The difference in the hydrophilic nature of LNPs obtained via the self-assembly approach might stem from the origin of the plant material, isolation approaches, and nanoparticle formation methods. During the organization of functional groups in LNPs, hydrophobic groups (phenylpropanoid units) tend to repel water and align inward on the particle’s surface. Conversely, the hydrophilic groups (mainly phenolic and aliphatic hydroxyl groups) are displayed on the outer shell, facilitating interactions with water.^23–28^ Therefore, nanoparticle formation through antisolvent precipitation results in hydrophilic LNPs. The lignin extraction method plays a critical role in determining both the size and the wettability of the resulting particles. The larger particle size of BB-LNPs may contribute to increased surface roughness, which, in turn, would influence the measured contact angle. Lignin precipitated from the kraft process (LB) undergoes a delignification procedure that typically results in the incorporation of sulfur and other residual components.^29^ In contrast, lignin extracted via the BLN process (BB), which employs pressurized hot water extraction followed by alkaline treatment (sodium hydroxide) for lignin isolation, is sulfur-free with reduced structural degradation compared to kraft lignin and therefore considered more chemically pure.^30^

In a previous study, the amounts of monomeric lignin units—guaiacyl (G) and syringyl (S)—were quantified for LB-LNPs and BB-LNPs to assess differences in their structural composition. Understanding the G/S ratio provides insight into the reactivity, polarity, and interfacial behavior of lignin nanoparticles, which can influence their performance in colloidal systems and in material applications. The guaiacyl content in LB-LNPs, derived from softwood, was 88.4%, with no syringyl units detected. These particles also contained 3.7% sulfur.^6^ This highlights the structural modifications induced by kraft pulping and the associated limitations in elucidating detailed self-assembly mechanisms. In contrast, BB-LNPs derived from hardwood showed a syringyl content of 63.4%, a guaiacyl content of 29.37%, and no detectable sulfur.^6^ The increased hydrophobicity observed in BB-LNPs is likely due to the presence of two methoxy groups in the syringyl unit, along with an absence of sulfur, although the exact role of the methoxy groups remains unclear. A higher S/G ratio contributes to a more linear structure due to fewer cross-linking sites on the aromatic ring, which may reduce π–π interactions but likely increases hydrophobicity.^31,32^

The interaction between LNPs and oil/water is relevant in many applications, though only a few studies have explicitly investigated them.^24^ The IFT curves of LNPs exhibited typical behavior seen with macromolecular emulsifiers, where the IFT continued to decrease until reaching a quasi-equilibrium state. This phenomenon occurs as macromolecular emulsifiers undergo conformational changes in their hydrophobic domains within the aqueous environment. The rearrangement continues as the emulsifier adsorbs at the interface and interacts with coadsorbing molecules, leading to the maturation of the interfacial layer.^16^ Both LB- and BB-LNPs (significantly more due to the increased amount of methoxy groups) reduce the IFT of the oil/water system. The IFT curve displays the more rapid adsorption of BB-LNPs on the oil droplet than LB-LNPs. The BB-LNPs may orient themselves at the interfaces more efficiently than LB-LNPs and reduce IFT. Additionally, a higher S/G ratio contributes to a more linear structure due to the absence of the C5 cross-linking position on the aromatic ring in syringyl units, which can reduce π–π interactions compared to guaiacyl units.^33^

In emulsions, stabilizers reduce IFT, form protective barriers to prevent coalescence, and increase medium viscosity to slow droplet movement.^34^ The type and molecular characteristics of stabilizers significantly influence droplet size by affecting their diffusion and adsorption at the interface.^17^ Low-molar-mass stabilizers tend to produce smaller droplets due to faster diffusion. LB-LNPs, formed from higher-molar-mass lignin, were smaller than the BB-LNPs. This may be influenced by stronger hydrophobic and π–π interactions during self-assembly, although the effect of molar mass alone cannot be excluded. Interestingly, smaller LNPs led to larger emulsion droplets, suggesting that larger stabilizer particles enhance the emulsion stability. This is due to higher adsorption energy, thicker interfacial layers providing steric hindrance, and bridging effects between droplets. Additionally, larger particles better reduce Ostwald ripening by lowering the IFT more effectively.

Global TSI demonstrated the stability of emulsions as well as their stability changes during storage (Figure 7), where a lower index indicates higher stability. The lower TSI value in BB-E indicates higher stability of BB-E compared to the LB-E. The lower IFT of the BB-E than LB-E shows that less force is needed to increase the surface area of the interface between two phases, favoring the formation of more stable emulsions by making it easier to disperse one phase into another. Additionally, the contact angle measurement is indicative of the adhesion forces between the liquid and the surface, which influence the emulsion’s ability to form stable interfaces between the dispersed phase and the continuous phase. Therefore, there is a likelihood of BB-LNPs reducing the droplet coalescence by repelling the water phase, which is confirmed by optical microscopy (Figure S4). Furthermore, the higher stability of BB-E compared to LB-E might be because LB-LNPs have a smaller size than BB-LNPs and a higher surface area-to-volume ratio that can lead to higher surface energy. This results in a greater tendency for aggregation and coalescence of the emulsion droplets. Moreover, BB-LNPs, which contain syringyl units, are less likely to form condensed structures formed during wood lignification, which may influence particle stability. However, a direct correlation between polymer branching and nanoparticle stability remains to be confirmed.

### Effect of Spontaneous Hemicellulose Adsorption

GX is composed of a xylose backbone with side chains that often include glucuronic acid, whereas GGM consists of a backbone of glucose and mannose units with side chains of galactose. During the isolation of hemicelluloses from wood, lignin and other components are often coextracted, with increasing quantity aligned with extraction temperature and duration. Generally, the lignin content in GX is higher than in GGM; however, it depends on the types of hemicellulose source, isolation, and treatment method.^3,11,12,17,35^ GGM extract has a higher molar mass. GX has more glucuronic acid groups, which increases the ζ-potential. GX is less branched and has a higher degree of acetylation. The reported degrees of acetylation are about 20–25% for the birch GX extract and 15–20% for the spruce GGM extract. This higher degree of acetylation can be attributed to the structural nature of xylose units, which are more readily acetylated than the mannose and glucose units found in GGM.^36^

Adsorption was evaluated indirectly by analyzing changes in physicochemical properties and compositional shifts, as recently published.^6^ Pyrolysis results revealed that hemicellulose adsorption was 1.2% for spontaneous adsorption and 2.5% for laccase-assisted lignin–hemicellulose hybrid LNPs.^6^ The results suggest that hemicelluloses physically adsorb onto the surface of LNPs through a combination of hydrogen bonding, electrostatic interactions, and potentially entropic contributions related to surface activity, which may be influenced by the residual lignin content in the hemicelluloses. This coating significantly enhanced the colloidal stability of the LNPs at acidic pH compared to that of uncoated LNPs. Spontaneous adsorption of hemicelluloses on the surface of LNPs reduces the average hydrodynamic diameter, likely because the hemicellulose coating prevents aggregation, particularly at acidic pH.^37^ The increase in the ζ-potential of functionalized LNPs with hemicellulose (GX > GGM) could be attributed to the presence of hydroxyl and acetyl groups in the hemicellulose, which cover the surface functional groups of LNPs. The reason for the higher increase in GX is likely due to its structural arrangement, which allows for better adsorption and a more uniform coating on the LNPs.^2^

Functionalization of LNPs with hemicelluloses generally increased their hydrophilicity. However, interestingly, when coating BB-LNPs with GX (higher lignin content),^35^ hydrophobicity was increased. This variation may be attributed to surface heterogeneity or differences in surface topography induced by the distinct molecular structures and interactions of GGM and GX with the LNP surfaces. GGM may have formed a more uniform and hydrophilic coating, leading to a lower contact angle due to its greater branching. In contrast, GX, being more linear and potentially less uniformly adsorbed, could have resulted in partial surface coverage or enhanced roughness, thereby increasing the contact angle. The increase in the hydrophobicity of BB-LNPs after functionalization with GX was a phenomenon that warranted further investigation.

The hardwood lignins with syringyl units may be more compatible with the acetylated GX. Acetylation makes the GX-coated BB-LNPs less polar and more resistant to water absorption. In relation to our findings, the hydrophobic index (galactose < glucose < arabinose < mannose < xylose < ribose < deoxyribose)^38,39^ may explain why the GX-coated BB-LNPs, containing higher lignin and acetylated groups, display an increased hydrophobicity due to their favorable interaction with lipophilic compounds in water, aligning with the higher hydrophobic surface fraction in GX.

Hemicelluloses demonstrate an efficient capacity to adsorb at emulsion interfaces and stabilize them.^40,41^ Based on the purity of the LNPs and hemicelluloses, there is always a fraction of hemicelluloses and lignins that may link with each other through covalent bonds, via phenylglycoside, benzylether, and γ (γ)-ester connections. These structures have an amphiphilic character, providing an appropriate polarity gradient that allows for interaction with both hydrophilic (water) and hydrophobic (oil) phases simultaneously. Consequently, they play an active role in reducing the IFT at the oil/water boundary.^11,12^ There is a greater IFT impact of functionalized LNPs with GX than with GGM, possibly caused by a higher lignin content in GX than GGM, compounded by more acid groups in GX, which decreases the ζ-potential more effectively than in GGM. On the other hand, the ζ-potential of LNPs with GX is lesser than that with GGM, which may influence the IFT by reducing the energy required to expand the interface.

In emulsions, LB-E and hybrid LB-E display a large droplet size and low stability, whereas the droplets of both BB-E and hybrid BB-E are relatively small (Figure 6). The volume average diameter D­[4,3] of the LB emulsions decreased after 16 days of storage. The unexpected decrease in the average size of droplets during storage suggests that a number of larger droplets may not have been included in the sampling for the analysis. BB-E and their hybrid variations exhibit a lower D­[4,3] compared to that of LB-E and their hybrids. Specifically, prepared BB-GGM-E shows a larger droplet size compared to BB-E and BB-GX-E. This might be due to the lower residual lignin content in the hemicellulose.^17^ This is similarly observed for GGM, where the reduced lignin content in hemicellulose could also contribute to the larger droplet size. Additionally, the different kinds of lignin moieties, carbohydrate composition, and molar mass are known to affect the aggregation tendency.^35^

The residual lignin content and composition in the hemicellulose, along with different molar mass and carbohydrate compositions, play significant roles in the stabilization of emulsions. The BB-GX-E droplet is smaller than the BB-GGM-E droplet, which might indicate a faster rate of adsorption. However, given sufficient time for adsorption, the rate might not be the critical factor. Instead, the lignin content and type could be crucial. It has been reported that lignin residues in GGM and GX significantly impact emulsion stability, revealing that GX has a higher emulsion stabilization capacity than GGM.^17^

The volume average diameter of emulsion droplets D­[4,3] is particularly sensitive to changes in the size of larger droplets (Figure S6a). Increasing the volume of BB-GGM-E caused instability, which could be due to the coalescence. For BB-E and BB-GX-E, the volume remained almost constant with slight aggregation, as shown in the optical microscopy image (Figure S4). An investigation into the D­[3,2] results was conducted to evaluate the droplet surface average diameter (Figure S6b), and the results support earlier discussions on stability.

Hemicelluloses have shown the capacity to absorb at emulsion and oil droplet interfaces. It has been reported that GX increases long-term emulsion stability by efficiently covering the droplet surface,^11,12^ which can be explained by a combination of factors including their composition, linear molecular structure, molar mass, acetyl content, degree of acetylation, and absolute ζ-potential. Additional evidence for the role of GX in stabilizing oil droplets comes from its ability to form interfacial layers due to its glucuronic acid side groups, whereas in GGM-stabilized emulsions, less galactosylated structures tend to concentrate at the interface while highly galactosylated structures remain in the continuous phase^42^ The linear structure of glucomannan might lead to a particle-like morphology with a moderate surface charge, reducing electrostatic repulsion and facilitating the adsorption of these polysaccharides at the interface.^43,44^ Surface modification of LNPs with GX reduces interfacial tension and is likely to be caused by adsorption at the oil–water interface through hydrogen bonding and hydrophobic interactions, forming a stabilizing layer. The adsorption of GGM on the surface of LNPs leads to a moderate contact angle, indicating that GGM can partially spread and attach to the oil–water interface. This reduces the interfacial tension between the oil and water by allowing GGM to form a stable layer around the droplets. As a result, this stable film helps to maintain the size of the droplets and prevents them from merging, which stabilizes the emulsion.

The study demonstrates the complex interplay among lignin composition, hemicellulose coating, particle size, and emulsion stability in LNPs. LB-LNPs, characterized by stronger π–π interactions and smaller particle size, exhibit higher hydrophilicity but lower emulsion stabilization potential. In contrast, BB-LNPs, with larger size and more hydrophobic syringyl-rich lignin, show improved emulsion stabilization, particularly when functionalized with GX. The coating of GX on BB-LNPs enhances the hydrophobicity and improves interface interactions, contributing to more stable emulsions. The differences in the ζ-potential and acetylation between GX and GGM further highlight their role in modulating the LNP behavior at oil–water interfaces. Overall, GX-coated BB-LNPs provide better emulsion stabilization, driven by their molecular structure, adsorption efficiency, and enhanced hydrophobic properties.

### Impact of Laccase-Induced Hemicellulose Adsorption

The impact of laccase treatment on the size and ζ-potential of LB-LNPs and BB-LNPs has been reported by Figueiredo et al.^3^ Laccase treatment increased the size of LB-LNPs due to oxidized dimer formation and intercross-linking, resulting in heterogeneous suspensions. BB-LNPs increased only slightly in size, remaining homogeneous because syringyl units and their two methoxy groups prevent extensive polymerization. Laccase treatment also made the ζ-potential more negative due to surface oxidation, affecting the dispersion stability. The treatment reduced the phenolic hydroxyl content through Cα=O oxidation, particularly in S-type monomers. Different S/G ratios in lignins influenced various simultaneous oxidation reactions, including side-chain fragmentation, condensation, polymerization, and Cα–OH oxidation.^3^

The role of fungal laccases in promoting a stronger association between hemicelluloses and LNPs was investigated. Laccase treatment facilitated oxidative coupling between lignin moieties present in the hemicelluloses and the LNP surface, as confirmed by the enriched lignin-derived pyrolysis products and changes in the monosaccharide composition. These findings suggest that both physical adsorption and enzymatic cross-linking contribute to the formation of stable hybrid nanoparticles.^6^

Increasing the hydrophobicity of laccase-treated LNPs (Figure 2) could be due to Ca-oxidation in BB-LNPs and decreasing phenolic hydroxyl groups in LB-LNPs and BB-LNPs,^3,4^ and the formation of quinone through the oxidation of a phenolic unit, which involves the removal of electrons and protons, resulting in the formation of a phenoxy radical. This radical can then undergo further reactions, leading to the formation of quinone structures.^45^ Quinones are a class of organic compounds derived from aromatic compounds, generally considered to be hydrophobic due to their aromatic ring structure and the presence of carbonyl groups. Laccase can act on phenolic and polymeric aromatic compounds on LNPs, such as Cα–OH oxidation, depolymerization, demethylation, and polymerization. After laccase treatment, reactive free radicals in substrates can lead to the formation of dimers and oligomers through various covalent linkages (C–C, C–O, and C–N bonds).^3,46^

Lignin polymerization during laccase treatment is influenced by reaction parameters such as enzyme activity and dosage, pH, initial lignin concentration, and temperature.^20^ Therefore, laccase treatment can lead to the polymerization of lignins, increasing their molar mass and changing their solubility. This process can result in a more hydrophobic lignin surface due to the formation of new C = C bonds between lignin units and the possible loss of hydrophilic groups. Increased hydrophobicity can lower the affinity of LNPs for the aqueous phase, increasing their tendency to migrate to and stabilize the oil/water interface. Our previous study suggested that there is a higher formation of Cα-oxidized syringyl units in BB-LNPs compared to that in the LB-LNPs rich in guaiacyl units. BB-L are more inert toward structural modification and polymerization using laccase treatment and more sensitive to oxidation.^3^ Hence, the hydrophobicity of LB-L is greater than that of BB-L, which might be mainly due to the higher amount of guaiacyl units driving the hydrophobicity, even in the absence of quinone formation.^43^

As laccase primarily targets phenolic substrates, GX is more susceptible to laccase treatment compared to GGM. Additionally, Cα-oxidation has been observed in BB-LNPs but not in LB-LNPs, which may explain the increased hydrophobicity of BB-LNPs.^3^ Furthermore, BB-GX-L results in greater quinone formation, making the substrate more hydrophobic than BB-GGM-L. The minimal variability in the IFT effects of different laccases on LB-LNPs (Figure 3) might be due to softwood lignins primarily consisting of guaiacyl units, which have fewer functional groups available for laccase action. The relative simplicity and lower content of reactive sites in softwood lignins may result in fewer opportunities for laccase-induced changes that affect the IFT. While the laccases increased the IFT of BB-LNPs, there were no differences based on the type of laccase used, possibly due to the uniform oxidation potential of the LNPs by the laccases.

There is a possibility that laccase treatment increased the IFT of BB-LNPs more than LB-LNPs due to the higher hydrophobicity of BB-LNPs.^24,47,48^ While increasing hydrophobicity typically enhances the adsorption of LNPs at the oil/water interface, leading to a reduction in interfacial tension and more stable emulsions, the observed increase in IFT suggests that laccase-treated BB-LNPs might be due to the quinone formation during laccase treatment altering the surface chemistry of BB-LNPs. While quinones increase hydrophobicity, they may also disrupt the amphiphilic balance necessary for effective adsorption at the interface. If too many hydrophobic groups are exposed, then the nanoparticles may fail to interact effectively with the water phase, leading to poor adsorption.

Laccase treatment did not affect the volume average diameter (D­[4,3]) of the LNPs and hybrid-LNP emulsions. The surface average diameter (D­[3,2]) of emulsions after laccase treatment remained the same after 16 days of storage, except for BB-GGM-L-E, which increased drastically after 16 days of storage and consequently did not stabilize the system, which might be due to coalescence and creaming. The emulsion stability data of LB-L-E, LB-GGM-L-E, and LB-GX-L-E show decreases in the TSI value, indicating increased stability. However, the emulsions remained unstable, potentially due to the oxidation of the phenolic hydroxyl groups, leading to a decrease in polar and hydrophilic functional groups (hydroxyl groups) in the structure. This reduction in the number of polar groups contributes to an increase in hydrophobic character and leads to aggregation, flocculation, and creaming, as confirmed by optical microscopy. The emulsion stability data of the laccase treatment display that BB-GX-L-E remained stable. This might be due to GX having a higher lignin content in the structure, allowing laccase to oxidize the phenolic hydroxyl group and form quinones, which cause higher hydrophobicity. GX, which is less branched than GGM, can cover the BB-LNPs more efficiently and increase the stability of the emulsion by covering the BB-LNPs. Laccase treatment oxidizes the phenolic hydroxyl groups, leading to a decrease in the number of polar and hydrophilic functional groups (hydroxyl groups) in the structure. This reduction in polar groups contributes to an increase in hydrophobic character. The increase in IFT causes instability in BB-L-E due to quinone formation, disrupting the amphiphilic balance. The increased hydrophobicity hinders adsorption, leading to a further increase in IFT and emulsion instability. The stability of BB-GX-L-E is likely due to effective interfacial coverage after laccase treatment, highlighting the key role of GX’s linear structure and molecular interactions in stabilizing the emulsion, while GGM’s branched structure may not provide the same level of stability.

Laccase treatment alters the surface properties of LNPs by inducing quinone formation and Cα oxidation, leading to increased hydrophobicity and reduced phenolic hydroxyl groups. This enhances the potential of LNPs for emulsification by promoting their migration to the oil/water interface. However, in BB-LNPs, the increased hydrophobicity following laccase treatment can disrupt the amphiphilic balance, resulting in reduced adsorption efficiency and emulsion instability, as shown by the increased IFT. In contrast, BB-GX-LNP emulsions remain stable due to the linear structure of GX, which provides better interfacial coverage than that of GGM. These results highlight the importance of lignin structure and surface chemistry in optimizing the use of laccase-treated LNPs for industrial applications.

## Conclusions

This study provides insights into the role of surface functionalization of LNPs derived from softwood and hardwood in influencing their interfacial functionality (solid–gas and liquid–liquid interfaces) and their performance in an oil/water system using Pickering emulsions. Key findings highlight that the inherent properties of LNPs, such as hydrodynamic diameter, surface charge, contact angle, and IFT, are substantially affected by the type of lignin source and the method of functionalization, including spontaneous hemicellulose adsorption and laccase-induced hemicellulose adsorption.

1. **Plain LNPs**: Softwood-derived LB-LNPs exhibited smaller hydrodynamic diameters and higher hydrophilicity compared to hardwood-derived BB-LNPs. This was attributed to the higher molar mass of LB lignin and stronger π–π interactions among guaiacyl units. Conversely, BB-LNPs displayed greater hydrophobicity due to syringyl units and associated methoxy groups, which led to a more significant reduction in interfacial tension (IFT) of hexadecane–water system and better emulsion stabilization.
2. **Spontaneous hemicellulose adsorption**: The spontaneous adsorption of hemicelluloses (GX and GGM) onto LNPs enhanced their hydrophilicity and stability in emulsions. GX, with higher lignin content and glucuronic acid groups, decreased the ζ-potential and increased the stability of emulsions more effectively than GGM. Interestingly, GX increased the hydrophobicity of BB-LNPs, a phenomenon warranting further investigation. Functionalization with hemicelluloses improved the emulsion stability by reducing the IFT and preventing aggregation.
3. **Laccase-induced hemicellulose adsorption**: Laccase treatment significantly impacted the structural and surface properties of LNPs, especially those derived from softwood. This treatment led to increased hydrophobicity due to the formation of quinone structures and increased interfacial stabilization properties. Laccase-treated LNPs showed better potential for industrial applications due to their altered hydrophobic and hydrophilic properties. Notably, the formation of quinone structures is more prevalent in BB-LNPs than in LB-LNPs, likely due to differences in lignin composition and reactivity

Overall, this study demonstrates that the type of lignin source, method of nanoparticle formation, and subsequent functionalization critically influence the interfacial behavior of LNPs. This understanding can guide the development of tailored LNPs for specific applications in food, pharmaceuticals, cosmetics, and biomedicine, where stable and efficient emulsions are essential. The observed variations in emulsion stability and droplet size underscore the importance of optimizing LNP properties to achieve the desired functional outcomes.

## Experimental Section

### Materials

Softwood kraft Lignoboost (LB) lignin was provided by Stora Enso (Finland), and hardwood birch lignin (BB) [*Betula L.*] was obtained via the BLN process from CH Bioforce Oy (Finland). The LB-derived lignin nanoparticles (LB-LNPs) had a number-average molecular weight (*M*_n_) of 1451 Da, a weight-average molecular weight (*M*_w_) of 20 225 Da, and a polydispersity index (*M*_w_/*M*_n_) of 13.9. In comparison, the BB-derived LNPs (BB-LNPs) had an *M*_n_ of 831 Da, an *M*_w_ of 13 150 Da, and an *M*_w_/*M*_n_ of 15.8.^3^ Spray-dried (sd) spruce galactoglucomannan (GGM) and birch glucuronoxylan (GX), with molar masses of approximately 2800 and 3600 g/mol, respectively,^17^ were obtained from Boreal Bioproducts (Finland) and Luke (Finland). The hemicelluloses (GGM and GX) were produced through the pressurized hot water extract (PHWE) method.^49^ Acetone (Sigma-Aldrich, Finland) and ethanol (EtOH; 99.5%; ALTIA, Rajamäki, Finland) were both HPLC grade and used without further purification. Citric acid monohydrates were acquired from Sigma-Aldrich (Finland). Poly-l-lysine (PLL) solution was acquired from Sigma-Aldrich at a concentration of 0.1% (w/v) and a molecular weight (*M*_w_) of 150,000–300,000 g mol^–1^. *n-*Hexadecane, with a purity greater than 99.0% for synthesis purposes, was procured from Merck (Darmstadt, Germany).

### Methodology

#### Preparation of LNPs

To prepare LNPs, the acetone nanoprecipitation approach was adapted from Farooq et al.^50^ Briefly, 2 g of technical lignins were dissolved in 200 mL of acetone/water 3:1 (v/v) mixture and stirred overnight, followed by their filtration using a glass microfiber filter (Whatman GF/F, pore size 0.7 μm). The obtained solution was rapidly poured into 400 mL of Milli-Q water under vigorous stirring for 1 h. Acetone was further removed by evaporation under reduced pressure at 40 °C to obtain the LNP dispersions. Finally, the LNP suspensions were centrifuged for 15 min at 50,000*g* and redispersed with Milli-Q water using ultrasonication (Branson digital sonicator) at a frequency of 20 kHz, 30% oscillation amplitude (100 W) for 60 s.

#### Heterologous Expression of Fungal Laccases

The basidiomycete laccases heterologously expressed in *Pichia pastoris* X-33 were (CcLcc9; GenBank accession No. BK004119),^51^ (OrLcc2-D206N named here OrLcc2Mut),^52^ (TpLccMut), (PrLcc2; GenBank accession No. CAI56705), and Dichomitus squalensis (DsLcc4; GenBank accession No. TBU29213). Heterologous expression of laccases was performed as described in previous work.^3^ The best laccase-producing transformants were selected by using 2,2′-azino-bis­(3-ethylbenzathiazoline-6- sulfonate) (ABTS)-plate assay. These selected transformants were cultivated in a complex liquid medium, and laccase expression was induced in a minimal medium with daily methanol addition for 5 days. The cultivation supernatant was collected, and the protease activity was inhibited. The cultivation medium was concentrated using Amicon ultrafiltration units (10 kDa cutoff) until the final volume was reduced to 3–5 mL. Laccase activity was spectrophotometrically monitored using 2,6-dimethoxyphenol as a substrate.^3,53^

#### Functionalization of LNPs, Spontaneous Hemicellulose Adsorption

For the spontaneous hemicellulose adsorption on LNPs, 10 mg/mL LNPs were diluted with 25 mM citric acid at pH 5 and mixed with hemicelluloses in the same buffer (the final concentration of hemicelluloses in the reaction was 25 mg/mL (after 2 x dilution)). After 24 h of incubation under gentle stirring (200 rpm) at room temperature and ambient air circulation, the samples were centrifuged (fiberlite 0.5, 50,000*g*) for 15 min and washed with Milli-Q H2O by centrifugation at 50,000*g* for 30 min.

#### Functionalization of LNPs, Laccase-Induced Hemicellulose Adsorption

To prepare the laccase-treated LNPs, 10 mg/mL LNPs were diluted with 25 mM citric acid pH 5 and mixed with hemicelluloses in the same buffer (the final concentration of hemicelluloses in the reaction was 25 mg/mL (after 2 × dilution)) and laccase at the concentration of 1000 nk/g of lignin. After 24 h of incubation under gentle stirring (200 rpm) and at room temperature and ambient air (O_2_) circulation, the samples were centrifuged (fiberlite 0.5, 50,000*g*) for 15 min, combined into one tube, and washed with Milli-Q H_2_O using centrifugation at 50,000*g* and for 30 min. The oxidized hybrid LNPs were redispersed with Milli-Q water for further analysis.

### Characterizations

#### Size and ζ-Potential

To measure the average hydrodynamic diameter, ζ-potential, and polydispersity index (PDI) of LNPs through dynamic light scattering (DLS), the Malvern Zetasizer Nano ZS instrument (Malvern Instruments Ltd., UK) was employed. To conduct these measurements, the samples were diluted in Milli-Q water to achieve a concentration of 500 μg/mL.

#### Contact Angle and Surface Morphology

Contact angle, θ (theta), quantifies the wetting behavior of a liquid (water) on a solid surface at the three-phase boundary. The contact angle measurements were performed by using a CAM 200 system (KSV Instruments Ltd., Finland). We used a frame interval of 20 ms and captured 4 frames, reporting the results after 60 s. All of the samples were measured in triplicate. The contact angle measurement was performed using the method described by Farooq et al.^20^, with slight modifications.

To prepare the spin-coated specimens for CA, the substrates used, silicon wafers, were cut into 1.5 cm × 1.5 cm square substrates, followed by plasma cleaning before the spin-coating process. Then, an anchoring layer of PLL was applied to the substrate at 2000 rpm for 90 s, followed by particle layer deposition using the same parameters. The PLL layer was added to facilitate the physisorption of particles. Three different concentrations (0.5, 1, and 1.5 mg/mL) of LNPs were then used to coat the silicon wafer. A second deposition of particles was applied by spin coating again under the same conditions. To estimate the surface coverage and homogeneity, NanoWizard 4 XP BioScience atomic force microscopy (Bio AFM-Bruker) was used. The PPP-NCSTAuD-10 cantilever, with a constant force of 1.2–29 N/m, tip radius of 10–15 μm, and resonant frequency of 76–263 kHz, was employed to analyze the surface morphology.

#### Interfacial Tension (IFT) Measurement

IFT measurements of different LNPs at the oil/water interface were made by using the reverse pendant droplet technique. The LNPs have an affinity for the oil/water interface, driving the adsorption of particles at this interface. This analytical approach allows for a comprehensive exploration of factors encompassing thermodynamic states, fluid interface structure, stabilizer adsorption/desorption, and molecular stabilizer characteristics.^50,54–58^ This behavior was quantified by experimentally measuring the adsorption isotherm of LNPs onto the oil/water interface.^59^

The reverse pendant drop method was performed on an optical tensiometer (CAM 200, KSV Instruments Ltd., Finland) to record the over time change of interfacial tension of a suspended hexadecane (an oil model) drop immersed in a solution of the emulsifier.^50,54–58^ Approximately 7–9 μL of n-hexadecane was dispensed into the emulsifier solution (0.05 mg/mL LNPs in water) with a gastight syringe equipped with a hook needle (syringe model C205M, needle gauge 22, Hamilton Company, Giarmata, Romania). The hexadecane was saturated with pure water prior to use to prevent changes in drop volume due to Ostwald ripening.^16^ The reverse syringe needle was positioned in the center of a poly­(methyl methacrylate) (PMMA) cuvette measuring 1 × 1 × 4.5 cm filled with the sample.

Subsequently, images were captured after releasing an inverted pendant droplet of n-hexadecane using a sealed syringe attached to a reverse needle with an external diameter of 0.717 mm. The measurements were conducted under the following conditions: 1000 frames at a frame interval of 20 ms, followed by 3600 frames at a frame interval of 1 s, resulting in a total of 4600 frames captured over 3620 s. The recorded drop shape was fitted by using CAM 200 software and the Young–Laplace model to determine the oil–water interfacial tension.

Measurements were performed for 60 min and 20 s (3620 s) at room temperature (approximately 21 ± 1 °C) with no evaporation compensation, as the oil layer formed by discarding the first few drops from the needle effectively prevented evaporation. Each sample was measured in triplicate, and the data was presented as a representative curve from one of the replicates. Since each replicate measurement involved a freshly dispensed drop and fresh dispersion, the interfacial tension values at each time point could not be averaged. The IFT value was read at the final time point of measurement using the representative curve from one of the replicates rather than averaging across replicates.

#### Emulsion Formulation, Characterization, and Stability

Preparation of Oil/Water Emulsion: the LNPs (0.5 wt %) were dispersed in 5 mM citric acid buffer, and the pH was adjusted to 7 using sodium hydroxide 0.1 molar. Next, 5 wt % *n-*hexadecane was added to the suspension, which was further mixed using an Ultra-Turrax (T18 basic, IKA, Staufen, Germany) equipped with a disperser-type stirrer, at 11,000 rpm for 1 min to form a coarse emulsion. To obtain a fine dispersion, the coarse emulsion was then subjected to ultrasonication, 40% oscillation amplitude (100 W), for 60 s (10 s on, 5 s off). Pictures were taken after the preparation of the emulsion to evaluate the physical appearance.

Emulsion Droplet Size and Morphology: the emulsion’s droplet size distribution was evaluated utilizing a Mastersizer Hydro 3000 SM (Malvern Instruments Ltd., Worcestershire, UK) employing a static light scattering technique. Refractive indices of 1.33 and 1.434 were applied for water and *n-*hexadecane, respectively. Before sampling, emulsions were gently inverted to have a homogenized sample, and samples were taken at the midway point of the emulsion volume. Three replicates were conducted for each measurement point: immediately after emulsion preparation (day 0) and at 16 days. The results are presented as droplet size distributions and surface-weighted average diameters of the emulsions. The average and standard deviation values of D­[3,2] were taken from two replicate measurements of three readings each. Droplet morphology was examined using optical light microscopy (DM IL LED, Leica Microsystems CMS GmbH, Wetzlar, Germany) just after emulsion preparation.

#### Emulsion Turbidity and Sedimentation Kinetics

The kinetic stability of emulsions throughout storage was tracked using a Turbiscan Lab Expert (Formulaction, Toulouse, France) with near-infrared light at a wavelength of 800 nm. Both transmitted and backscattering light intensities were amalgamated using Turbisoft version 1.2 (Formulaction, Toulouse, France) software to determine the Turbiscan Stability Index (TSI) of the emulsions. The outcomes were expressed in terms of the overall TSI, combining partial results from the lower third (bottom), central third (middle), and upper third (top) portions. Measurements were conducted immediately after emulsion preparation, on day 7, and on day 16 of storage.

## Supplementary Material



## Notes

Dynamic light scattering characterization of LNPs (size, ζ-potential), AFM image of LNP deposition on silicon wafers, interfacial tension measurements of LNPs with and without laccase treatment, optical microscopy images of emulsions, emulsion stability over 16 days, and volume- and surface-average diameters [D­(4,3), D­(3,2)] of emulsions (PDF)

## Abbreviations

BB: Birch lignin (hardwood lignin)
E: emulsion
GGM: galactoglucomannan (softwood hemicellulose)
GX: glucuronoxylans (hardwood hemicellulose)
L: laccase-treated
LB: Lignoboost lignin (softwood lignin)
LNPs: lignin nanoparticles

## Colloidal Lignin Nanoparticles

BB-GGM: BB-LNPs functionalized with GGM (spontaneous adsorption)
BB-GGM-L: BB-LNPs functionalized with GGM (laccase-treated)
BB-GX: BB-LNPs functionalized with GX (spontaneous adsorption)
BB-GX-L: BB-LNPs functionalized with GX (laccase-treated)
BB-L: laccase-treated BB-LNPs
BB-LNPs: Birch lignin nanoparticles
LB-GGM: LB-LNPs functionalized with GGM (spontaneous adsorption)
LB-GGM-L: LB-LNPs functionalized with GGM (laccase-treated)
LB-GX: LB-LNPs functionalized with GX (spontaneous adsorption)
**LB-GX**-L: LB-LNPs functionalized with GX (laccase-treated)
LB-L: laccase-treated LB-LNPs
LB-LNPs: Lignoboost lignin nanoparticles

## Emulsions

BB-E: emulsion of BB-LNPs
BB-GGM-E: emulsion of BB-LNPs functionalized with GGM (spontaneous adsorption)
BB-GGM-L-E: emulsion of BB-LNPs functionalized with GGM (laccase-treated)
BB-GX-E: emulsion of BB-LNPs functionalized with GX (spontaneous adsorption)
BB-GX-L-E: emulsion of BB-LNPs functionalized with GX (laccase-treated)
BB-L-E: emulsion of laccase-treated BB-LNPs
LB-E: emulsion of LB-LNPs
LB-GGM-E: emulsion of LB-LNPs functionalized with GGM (spontaneous adsorption)
LB-GGM-L-E: emulsion of LB-LNPs functionalized with GGM (laccase-treated)
LB-GX-E: emulsion of LB-LNPs functionalized with GX (spontaneous adsorption)
LB-GX-L-E: emulsion of LB-LNPs functionalized with GX (laccase-treated)
LB-L-E: emulsion of laccase-treated LB-LNPs
