# Supplementary material for: Interfacial Properties of Surface-Modified Lignin Nanoparticles
Source: Langmuir. 2025 Jul 4;41(27):17654–67. doi: 10.1021/acs.langmuir.5c01244 (PMC12269062; doi:10.1021/acs.langmuir.5c01244)
Supplement: Supplementary file 1 [file la5c01244_si_001.pdf]

Interfacial properties of surface-modified lignin nanoparticles

Mina Zare[1,2,*], Patrícia Figueiredo[1], Maarit H. Lahtinen[1], Kristiina S. Hilden[2,3], Sami Hietala[4], Kirsi S. Mikkonen[1,2,*]
[1] Department of Food and Nutrition, Faculty of Agriculture and Forestry, University of Helsinki, 00014 Helsinki, Finland
[2] Helsinki Institute of Sustainability Science (HELSUS), University of Helsinki, 00014 Helsinki, Finland
[3] Department of Microbiology, Faculty of Agriculture and Forestry, University of Helsinki, 00014 Helsinki, Finland
[4] Department of Chemistry, University of Helsinki, 00014 Helsinki, Finland
Corresponding authors Email ID: kirsi.s.mikkonen@helsinki.fi , mina.zarelahti@helsinki.fi

Supporting information

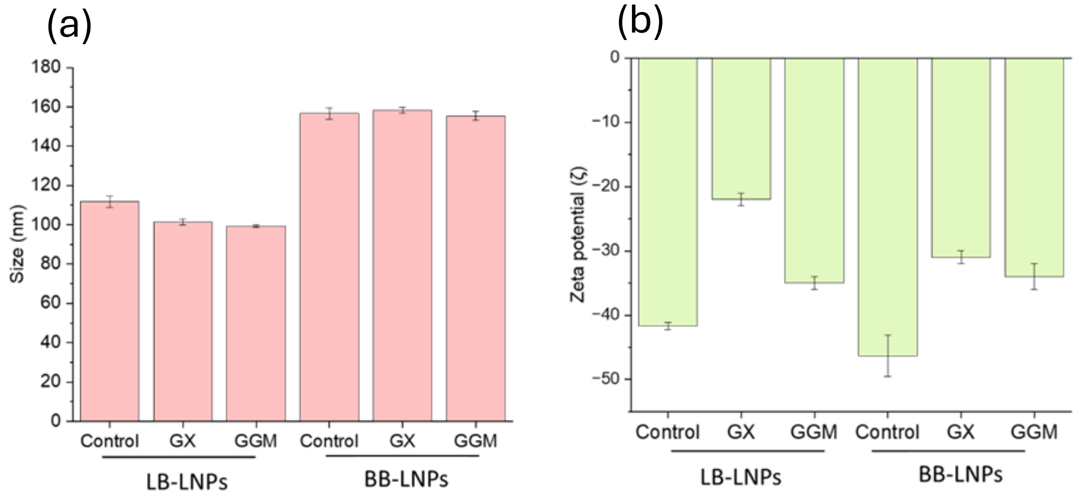

Figure S1. characterization of LNPs by dynamic light scattering in terms of (a) size, and (b) ζ-potential. Refer to Table 1 for abbreviations.

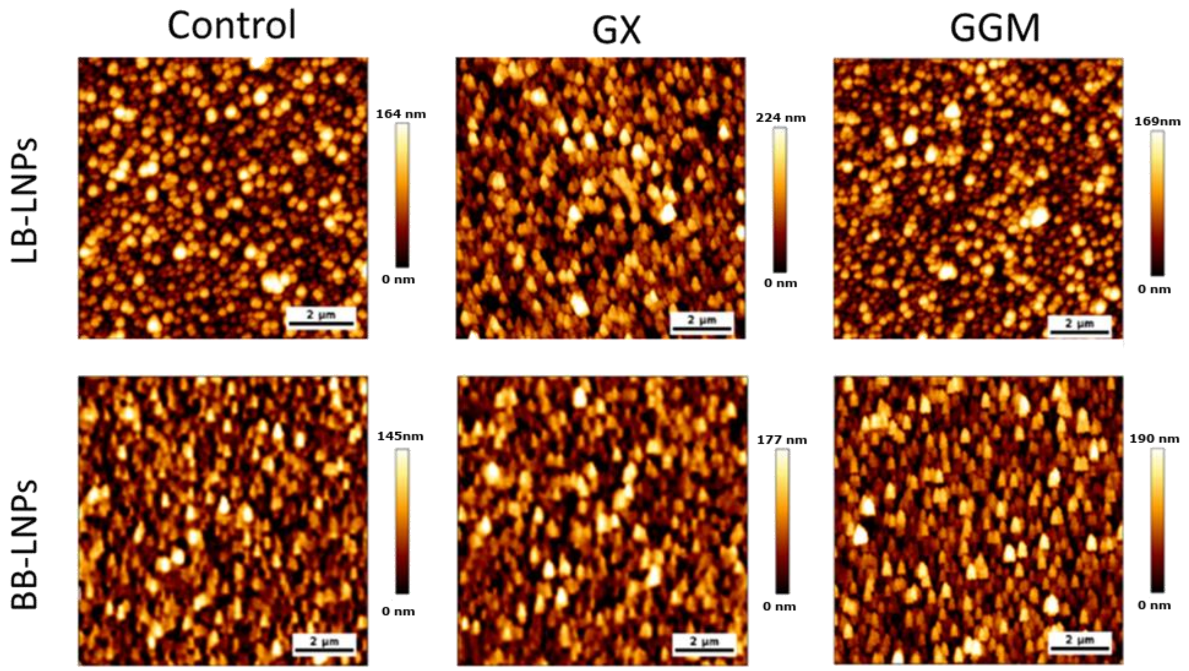

Figure S2. image of 1mg/ml double layer deposition of LNPs on the surface coverage of silicon wafers (1.5×1.5 cm) by atomic force microscopy. Refer to Table 1 for abbreviations.

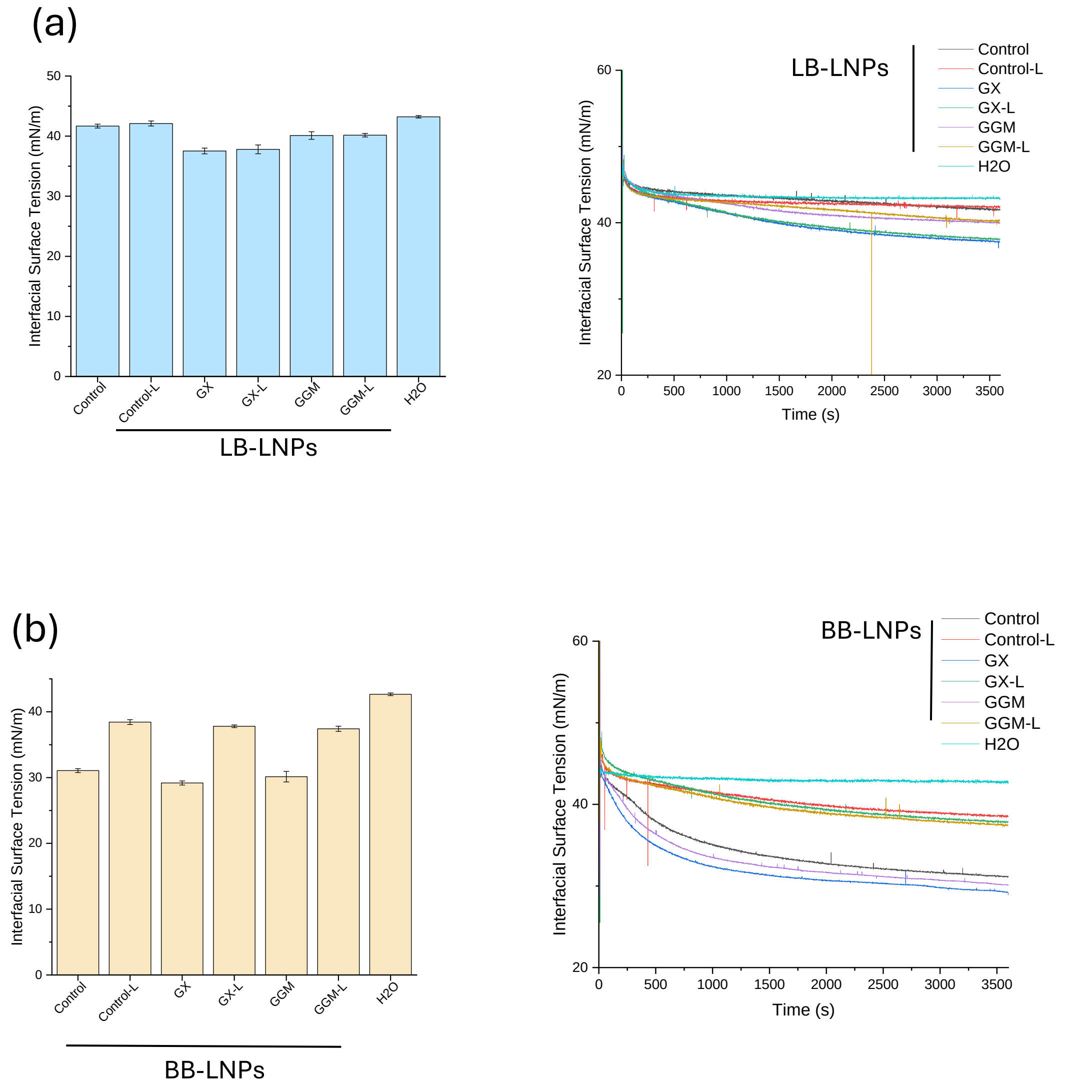

Figure S3. interfacial tension of (a) LB-LNPs and (b) BB-LNPs and their hybrid hemicellulose-LNPs over 3600 seconds, before and after *Ds*Lcc4 laccase treatment. Refer to Table 1 for abbreviations.

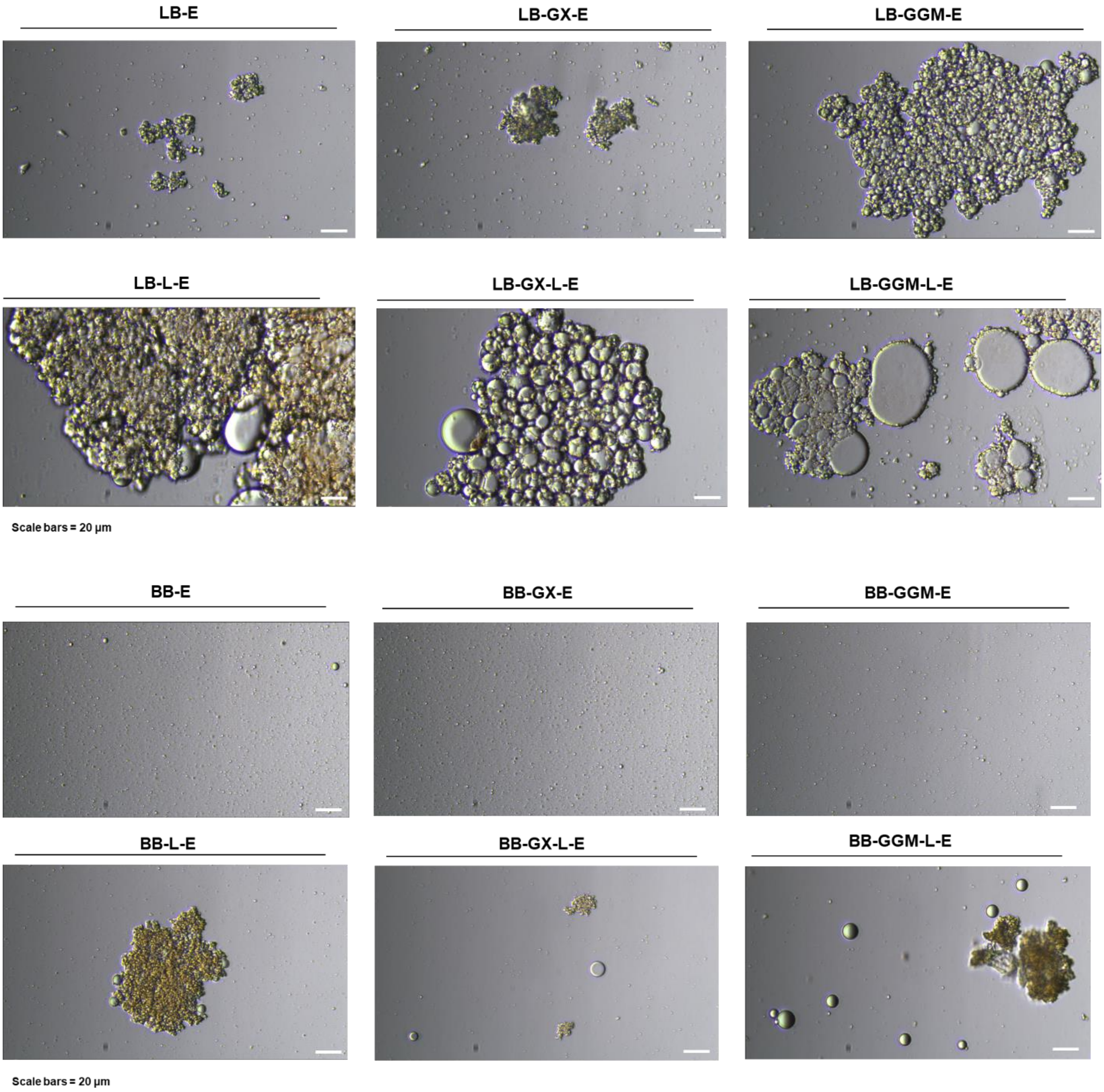

Figure S4. optical microscopy image of the emulsion before and after laccase treatment. Refer to Table 1 for abbreviations.

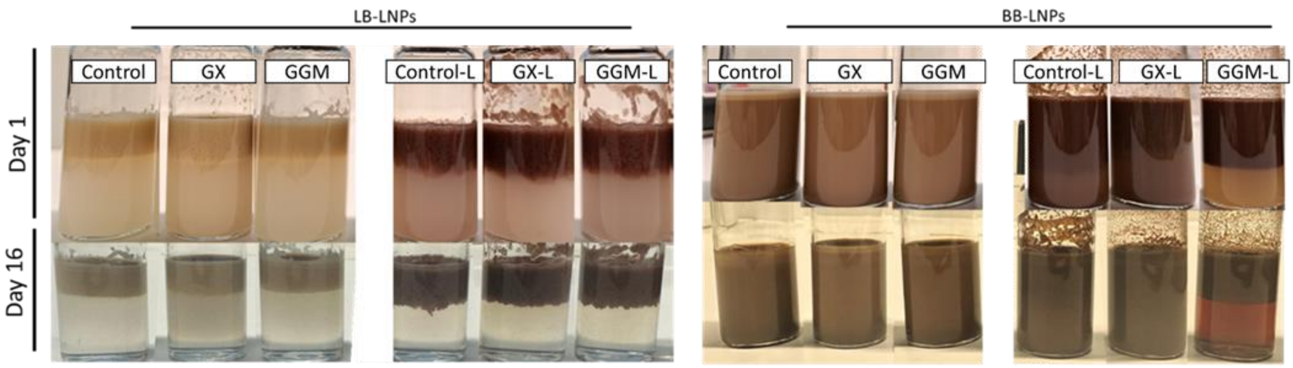

Figure S5. displays the emulsion stability of functionalized LNPs on day 0 and after 16 days of storage. Refer to Table 1 for abbreviations.

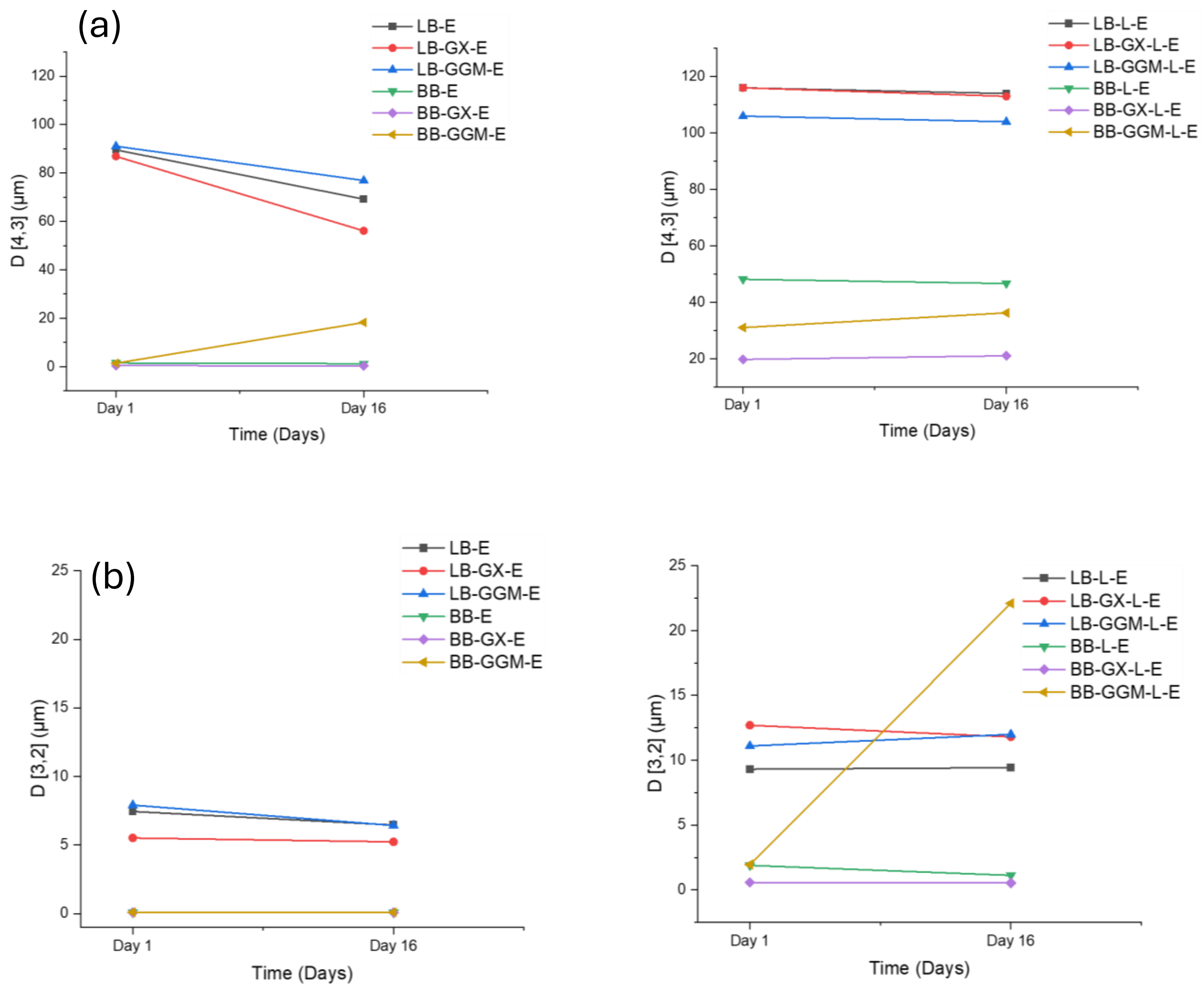

Figure S6 (a) represents the volume-average diameter [D(4,3)], and (b) displays the surface-average diameter [D(3,2)] on days 0 and 16 for stabilized emulsions. Refer to Table 1 for abbreviations.
